# Human ASPM participates in spindle organisation, spindle orientation and cytokinesis

**DOI:** 10.1186/1471-2121-11-85

**Published:** 2010-11-02

**Authors:** Julie Higgins, Carol Midgley, Anna-Maria Bergh, Sandra M Bell, Jonathan M Askham, Emma Roberts, Ruth K Binns, Saghira M Sharif, Christopher Bennett, David M Glover, C Geoffrey Woods, Ewan E Morrison, Jacquelyn Bond

**Affiliations:** 1 Section of Ophthalmology and Neuroscience, Wellcome Trust Brenner Building, Leeds Institute of Molecular Medicine, University of Leeds, St. James's University Hospital, Leeds LS9 7TF, UK; 2 Department of Life Sciences, The Open University, Walton Hall, Milton Keynes, MK7 6AA, UK; 3 Cancer Research UK Cell Cycle Genetics Research Group, University of Cambridge, Department of Genetics, Downing Street, Cambridge CB2 3EH, UK; 4 CRUK Clinical Centre at Leeds, Division of Cancer Medicine Research, Leeds Institute of Molecular Medicine, St James's University Hospital, Leeds LS9 7TF, UK; 5 Yorkshire Regional Genetics Service, Yorkshire Regional Genetics Service, Ashley Wing, St James's University Hospital, Leeds LS9 7TF, UK; 6 Cambridge Institute for Medical Research, Wellcome Trust/MRC Building, Addenbrooke's Hospital, Hills Road, Cambridge, CB2 2XY, UK

## Abstract

**Background:**

Mutations in the Abnormal Spindle Microcephaly related gene (*ASPM) *are the commonest cause of autosomal recessive primary microcephaly (MCPH) a disorder characterised by a small brain and associated mental retardation. ASPM encodes a mitotic spindle pole associated protein. It is suggested that the MCPH phenotype arises from proliferation defects in neural progenitor cells (NPC).

**Results:**

We show that ASPM is a microtubule minus end-associated protein that is recruited in a microtubule-dependent manner to the pericentriolar matrix (PCM) at the spindle poles during mitosis. *ASPM *siRNA reduces ASPM protein at the spindle poles in cultured U2OS cells and severely perturbs a number of aspects of mitosis, including the orientation of the mitotic spindle, the main determinant of developmental asymmetrical cell division. The majority of ASPM depleted mitotic cells fail to complete cytokinesis. In MCPH patient fibroblasts we show that a pathogenic *ASPM *splice site mutation results in the expression of a novel variant protein lacking a tripeptide motif, a minimal alteration that correlates with a dramatic decrease in ASPM spindle pole localisation. Moreover, expression of dominant-negative ASPM *C*-terminal fragments cause severe spindle assembly defects and cytokinesis failure in cultured cells.

**Conclusions:**

These observations indicate that ASPM participates in spindle organisation, spindle positioning and cytokinesis in all dividing cells and that the extreme *C*-terminus of the protein is required for ASPM localisation and function. Our data supports the hypothesis that the MCPH phenotype caused by *ASPM *mutation is a consequence of mitotic aberrations during neurogenesis. We propose the effects of *ASPM *mutation are tolerated in somatic cells but have profound consequences for the symmetrical division of NPCs, due to the unusual morphology of these cells. This antagonises the early expansion of the progenitor pool that underpins cortical neurogenesis, causing the MCPH phenotype.

## Background

During neurogenesis the majority of neurons and glia in the mammalian neocortex arise from the division of NPC in the neuroepithelial lining of the central cavities of the brain [1]. Primary NPC have a specific pattern of mitotic activity. Initially each symmetrical division increases precursor cell number by generating two progenitor cells per division. Subsequent asymmetric neurogenic divisions produce one neuron and regenerate one progenitor cell [2]. In the developing mammalian cortex the division fate of a cell appears dependent upon the orientation of the mitotic spindle and hence the position of the cleavage furrow with respect to the apical surface of the neuroepithelium [3]. As a result of the inheritance of cell lineage determinants located at the apical cell membrane, cleavage parallel to the apical surface results in neurogenic division where the apical contents are inherited by one daughter cell and the basal contents by the other, whereas perpendicular cleavage produces two daughter progenitor cells. The mechanisms regulating spindle orientation and cleavage furrow positioning in the mammalian neuroepithelium are not well understood.

Autosomal recessive primary microcephaly (MCPH) is a rare Mendelian disorder characterized by a congenital deficiency of foetal brain growth, particularly affecting the neocortex. This results in the formation of a small but structurally normal brain and associated mental retardation but no other neurological defects [4,5]. The concept that MCPH is a primary disorder of neurogenic mitosis, the result of which is a reduction of cell number in the developing human brain, is an attractive one.

Mutations that cause the condition have been found in five genes: microcephalin (*MCPH1*), which functions in the DNA damage response pathway; and abnormal spindle-like microcephaly associated gene (*ASPM*), CDK5 regulatory subunit-associated protein 2 (*CDK5RAP2*), centromeric protein J (*CENPJ*) and SCL/TAL1-interupting locus (*STIL*) which are all associated with aspects of centrosome function [5-13]. The most common cause of MCPH is mutation of the *ASPM *gene [5,14,15] at the *MCPH5 *locus on chromosome 1q31 [16,17]. All known pathogenic mutations produce a single clinical phenotype [5,15] even though they include nonsense, frameshift, translocation and splice site mutations located throughout the 28 exon *ASPM *gene [5,8,15,18-23]. It was originally assumed that mutations result in either protein truncation or mRNA degradation via the nonsense mediated decay (NMD) pathway [24].

**Figure 1:**
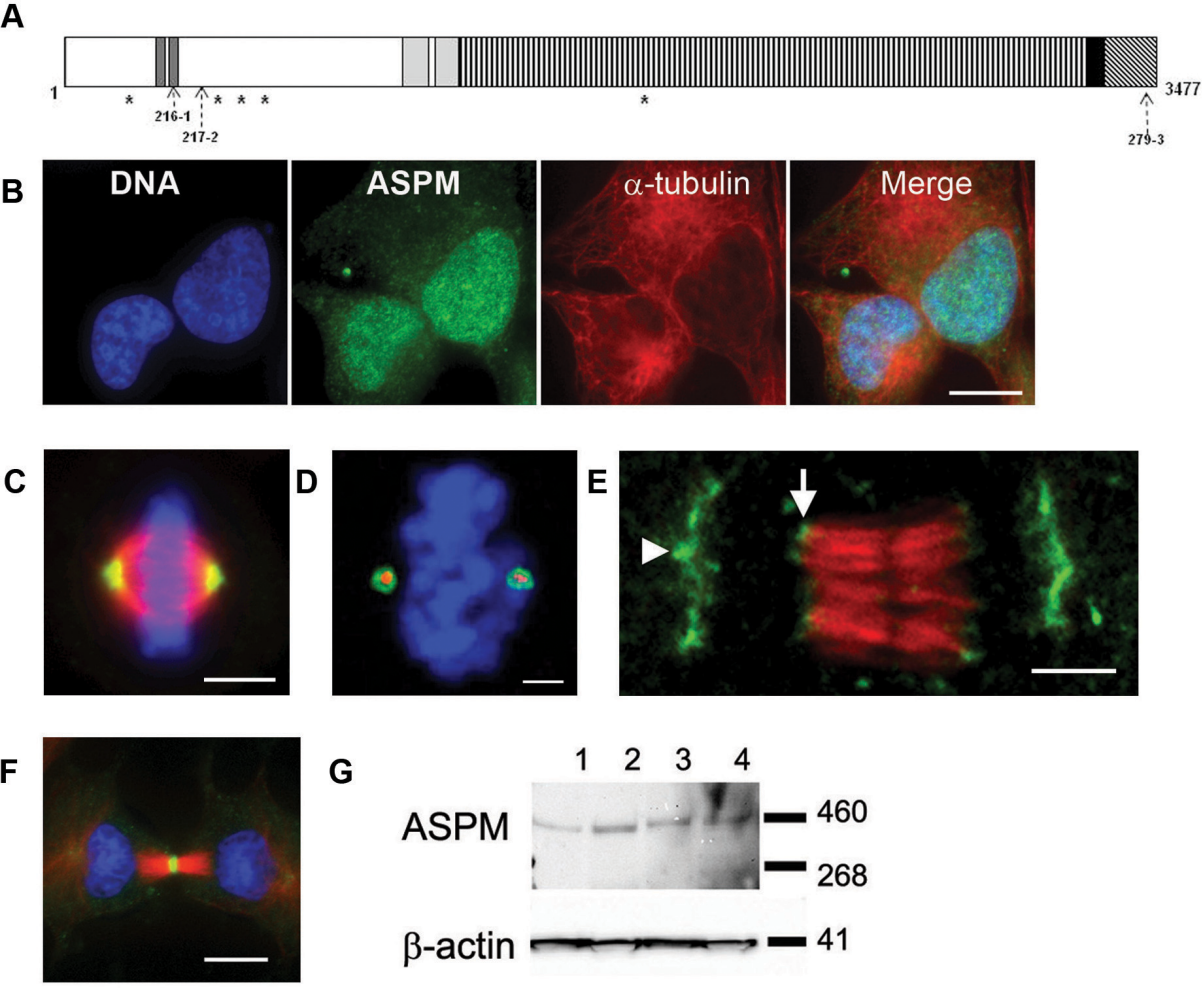
**Organisation of the ASPM protein and its cellular distribution during mitosis**. **A**. Structure of the 3477 amino acid human ASPM protein. The region corresponding to the microtubule-binding domain of *Drosophila *Asp [24] is shown in white. The calponin homology domains (aa920-1261) are in light grey; ASNP repeats are dark grey boxes (aa316-342, 366-400); 81 IQ domains (aa1267-3225) are shown as vertical stripes; the armadillo repeat-like domain (aa3294-3327) is a black box; and the *C*-terminal region is depicted by diagonal stripes. The location of potential nuclear localization sequences are indicated by asterisks and the location of the peptides used to raise polyclonal antibodies, by dashed arrows. **B-F**. Analysis of ASPM distribution following immunostaining. HeLa cells were fixed and stained with antibodies specific for ASPM (green), anti-α-tubulin (red) and with DAPI (blue) to identify nuclei. Panels B-E utilised Anti-ASPM 216-1, whilst anti-ASPM 279-3 was used in panel F. **B**. ASPM is predominantly nuclear in interphase cells. Scale bar = 10 μm. **C**. ASPM is localised to the spindle poles during metaphase. Scale bar = 5 μm. **D**. A globular distribution of ASPM (green) is seen around the γ-tubulin (red) immunopositive core of metaphase HeLa cell spindle poles. DNA (DAPI staining) is shown in blue (confocal image). Scale bar = 2.5 μm. **E**. Single 0.5 μm confocal section through the centre of a telophase HeLa cell immunostained to reveal ASPM (green) and α-tubulin (red). In addition to broad spindle pole-associated labelling (arrowhead), ASPM also localizes to the minus ends of central spindle microtubules (arrow). Scale bar = 2.5 μm. **F**. A late telophase fibroblast immunostained with anti-ASPM 279-3 (green), anti-α-tubulin (red) and with DAPI (blue). ASPM is predominantly localized at the midzone of the central spindle. Scale bar = 10 μm. **G**. Immunoblotting of cell lysates of COS7 cells (lane 1), U2OS cells (lane 2), primary fibroblasts (lane 3) and HeLa cells (lane 4) with anti-ASPM 217-2 antibody. This identifies a protein of approximately 410 kDa in each lane. A blot stained with anti-β-actin is shown as loading control.

The human *ASPM *gene encodes a protein composed of 3477 amino acids [5] that is predicted to contain an amino terminal microtubule binding domain [25]; two highly conserved N-terminal short ASNP (ASPM N-proximal) repeats [8]; two calponin homology domains; up to 81 calmodulin binding isoleucine-glutamine (IQ) motifs [5,8,26,27]; an armadillo-like sequence and a carboxyl terminal region of unknown function [5,15]. Figure 1A summarizes the domain structure of the ASPM protein. ASPM is expressed in all proliferating tissues and is upregulated in many cancers [8].

Human *ASPM *is the orthologue of the *Drosophila *abnormal spindle gene (*asp*). Asp is involved in spindle microtubule organisation in mitosis and meiosis [25,28-31] and in cytokinesis [32,33]. In dividing *Drosophila *neuroblasts *asp *mutations cause metaphase arrest, resulting in reduced CNS development [29]. siRNA depletion of *asp *produces a severe loss of microtubule focus at spindle poles [34]. In mice, elimination of *Aspm *results in a reduction in neural stem cell proliferation and increases the likelihood that NPCs will undergo asymmetric cell division, implying a reduction in the total number of progenitor cells formed during brain development [35]. Inhibition of human ASPM expression by siRNA-mediated knockdown inhibits tumour cell proliferation [36]. Although it has been hypothesized that ASPM is required for cell division in the developing human brain, direct evidence in support of this has been lacking.

In the present study we investigate the role of ASPM in human cell division and extend the characterization of the mitotic distribution of human ASPM. We show that *ASPM *knockdown in U2OS osteosarcoma cells by siRNA alters the positioning of the mitotic spindle from parallel to the substrate to perpendicular to the substrate, effectively altering the division symmetry from symmetrical to asymmetrical. *ASPM *siRNA-mediated depletion also induced cytokinesis failure and apoptosis. Moreover we show that a pathogenic mutation identified in an MCPH patient, and located in the 3'region of the *ASPM *gene instigates the activation of an upstream in frame cryptic splice donor site, resulting in the splicing out of nine nucleotides (nt) from the *ASPM *sequence. The resultant protein exhibits decreased efficiency in localization to spindle poles, suggesting the presence of the *C*-terminal domain is of critical importance for ASPM function. Supporting this, we find that ASPM *C*-terminal fragments expressed in transfected cells induce dominant-negative defects on spindle organisation and cytokinesis. This study provides further evidence for the function of ASPM in the division cycle of mammalian cells. We discuss these findings in relation to cerebral cortex neurogenesis and microcephaly.

## Results and Discussion

### ASPM is a nuclear protein that relocates to the spindle pole matrix and central spindle MT minus ends during mitosis

To determine the intracellular distribution of human ASPM, rabbit polyclonal antibodies were raised against ASPM-specific peptide sequences (Figure 1A) and used to screen a panel of cell lines by immunofluorescence. Two antibodies against *N*-terminal peptides (216-1 and 217-2) and one antibody against a *C*-terminal peptide (279-3) produced virtually identical staining patterns (see Additional file 1, 2, 3) in a variety of human cell types including HeLa, primary human dermal fibroblasts (HDF), U2OS osteosarcoma cells, SH-SY5Y neuroblastoma cells and in COS-7 African green monkey kidney cells. Pre-incubation with the associated ASPM peptide negated the ASPM signal (data not shown). As previously published [37], we found ASPM expression to be predominantly concentrated in the nucleus during interphase (Figure 1B), however we did not find evidence of centrosomal distribution. Consistent with this finding, the *ASPM *sequence contains a number of potential nuclear localization sequences (Figure 1A). No specific association with microtubules or microtubule-organizing centres was observed prior to nuclear envelope breakdown. In agreement with previous reports, ASPM immunoreactivity shifted away from condensing DNA and onto spindle structures immediately after nuclear envelope breakdown, and from prometaphase to anaphase ASPM was seen almost exclusively associated with the mitotic spindle (Figure 1C) [8,37,38]. We localized mitotic ASPM immunoreactivity to pole-proximal regions of spindle microtubules, where it formed a ring around the spindle poles. This ring enclosed but did not extensively overlap with γ-tubulin immunoreactivity (Figure 1D), though it co-localised with Dynactin 1 (DCTN1) immunoreactivity in the pericentriolar matrix (PCM, data not shown). In anaphase cells the mitotic spindle has flattened and ASPM maintains its spindle pole localisation, was observed along the microtubules leading to the pole and a fraction of ASPM was found at the minus ends of central spindle microtubules (Figure 1E). Throughout telophase and cytokinesis ASPM was predominantly positioned in a narrow ring at the centre of the midbody (Figure 1F and see Additional file 4). This localisation was most visible when using the *C*-terminal antibody 279-3.

Immunoblot analysis using the 217-2 *N-*terminal anti-ASPM antibody identified a 410 kDa ASPM band in each of a panel of human and monkey cell lines (Figure 1G). This band corresponds to the expected size of the 3447 amino acid wild-type ASPM protein. Other investigators have reported that at least three predominant ASPM splice variants may exist [8]. In accordance with the reported literature, 217-2 would be predicted to recognise wildtype ASPM and the largest variant V1, however we saw no evidence for the existence of V1 in our whole cell extracts. We were unable to optimise antibodies 216-1 and 279-3 for Western blotting at endogenous levels of ASPM. However, these antibodies recognise recombinant forms of the ASPM protein expressed in and purified from bacteria (results not shown).

### ASPM distribution is microtubule dependent

**Figure 2:**
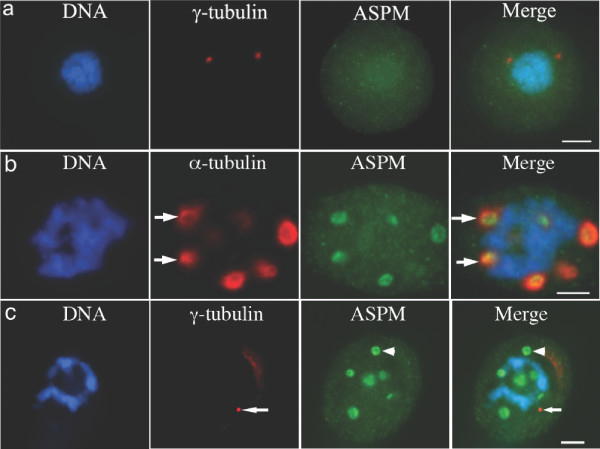
**The ASPM spindle association is microtubule-dependent**. **A**. Panel showing a nocodazole-treated mitotic HeLa cell immunostained for ASPM 216-1 (green), γ-tubulin (red) and DAPI (blue). ASPM is not clustered around the γ-tubulin of the spindle poles in the absence of microtubules. Scale bar = 5 μm. **B**. Panel showing a taxol-treated mitotic HeLa cell immunostained for ASPM 216-1 (green), α-tubulin (red) and DAPI (blue). ASPM concentrates in the centre of taxol-induced microtubule asters (arrows). Scale bar = 5 μm. **C**. Panel showing a taxol-treated mitotic HeLa cell immunostained for ASPM 216-1 (green), γ-tubulin (red) and DAPI (blue). ASPM (arrowhead) does not specifically co-localize with γ-tubulin foci (arrow) in taxol-treated cells. Scale bar = 5 μm.

To examine the dependence of ASPM localization upon microtubules, we first treated HeLa cells with 5 μg/ml of the microtubule depolymerising drug nocodazole for 60 minutes. Under these conditions γ-tubulin immunostaining was maintained at spindle poles but ASPM 216-1 staining was lost, indicating that the mitotic distribution of ASPM is microtubule-dependent (Figure 2A). We next challenged HeLa cells with 10 μM taxol for 60 minutes, a treatment that induces the formation of multiple microtubule asters in mitotic cells. ASPM 216-1 immunostaining was observed at the centre of these asters (Figure 2B), suggesting a specific localization to microtubule minus ends. ASPM was not specifically associated with the anastral γ-tubulin foci also seen in taxol-treated cells (Figure 2C).

### SiRNA mediated ASPM knockdown leads to repositioning of the mitotic spindle and cytokinesis dysfunction

**Figure 3:**
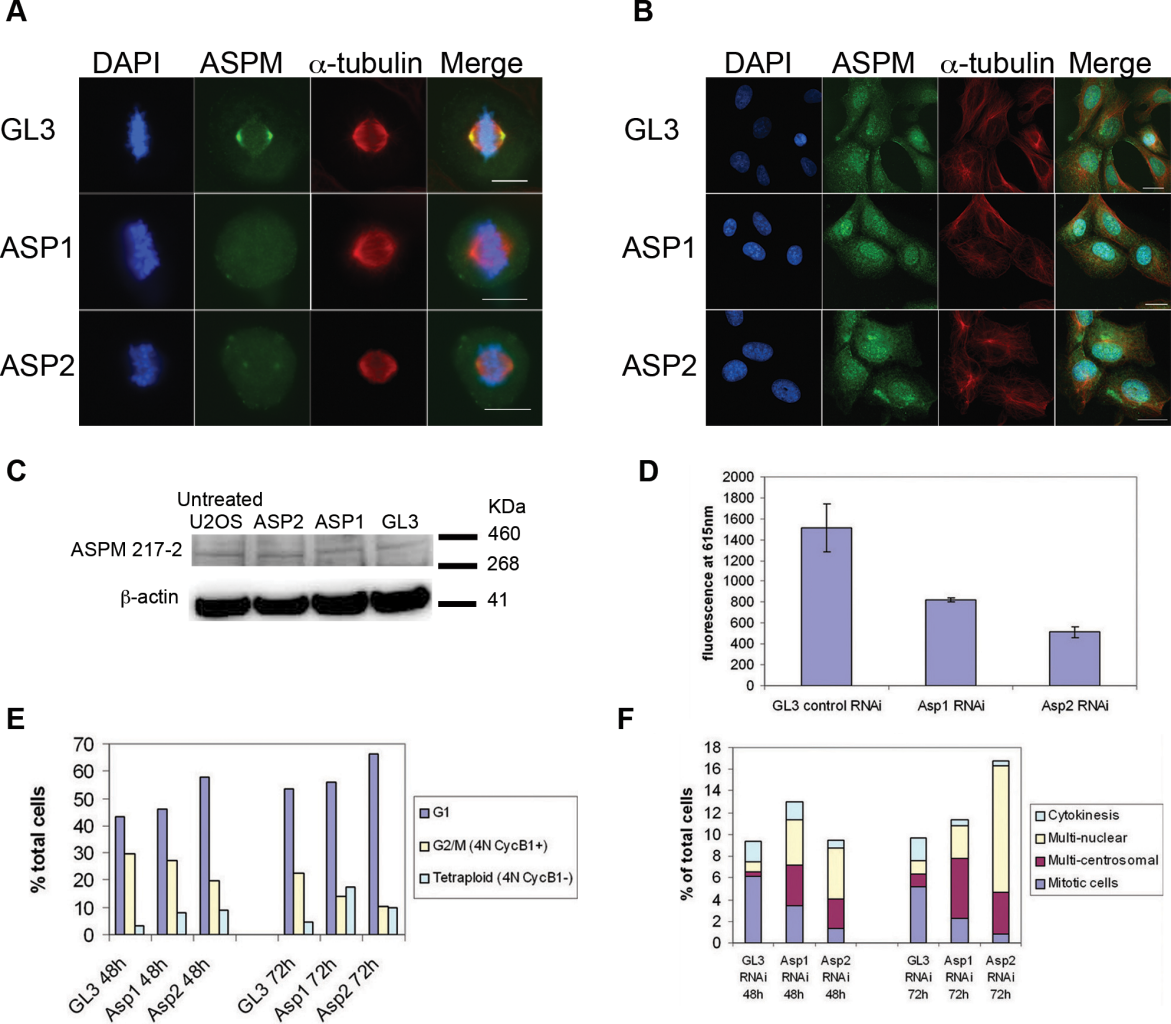
**Phenotypic traits of siRNA mediated depletion of ASPM in U2OS cells**. **A**. Reduction in spindle pole associated ASPM (using the anti-ASPM 216-1 antibody), 72 hrs post transfection with ASP1 and ASP2 siRNAs relative to GL3 control dsRNA Scale bar = 10 μm. **B**. ASPM (216-1 antibody) expression in interphase cells 72 hrs post GL3, ASP1 and ASP2 transfection. Scale Bar = 10 μm. **C**. Western Blot using rabbit polyclonal anti-ASPM 217-2 antibody, showing ASPM expression in lysates from GL3, ASP1 and ASP2 treated U2OS cells. A β-actin loading control is shown. 25 μg protein for each sample was loaded. **D**. Celltitre Blue cell viability assay was carried out 72 hours post siRNA treatment, showing a reduction in cell viability in ASP1 and ASP2 siRNA transfected cell cultures. **E**. Flow cytometric analysis of DNA content and cyclin B1 status 48 and 72 hours post ASPM siRNA treatment, demonstrating a decrease in the number of cells in G2/M and an increase in the number of tetraploid G1 cells. **F**. Analysis of immunofluorescently labelled cells treated for 48 and 72 h with ASP1 and ASP2 siRNAs. A decrease in mitotic index and an increase in the number of multinucleate cells and cells with centrosome numbers greater than 2 are seen following ASPM knockdown. A decrease in the number of cells displaying morphological hallmarks of cytokinesis, such as the presence of a midbody, is also seen. These observations are consistent with a failure to complete cytokinesis.

To determine the effects of loss of ASPM function upon cell cycle progression we carried out *ASPM *RNA interference experiments. As a normal human NPC culture amenable to RNAi studies was not available we chose to perform these tests in the extensively characterised and readily transfectable U2OS osteosarcoma cell line using two individual ASPM siRNAs (ASP1 and ASP2) and a luciferase (GL3) control. A clear reduction in ASPM at the spindle poles of dividing cells was detected after transfection with ASP1 and ASP2 for 72 or 96 hours. By 72 hours post transfection with ASP1, ASPM (detected with the anti-ASPM 216-1 antibody at the spindle poles of mitotic cells was detectable in only 24% (6/25) of metaphase cells and completely knocked down in 76% of dividing cells, whereas ASP2 siRNA achieved a partial knockdown in 92% (23/25) metaphase cells (Figure 3A) and complete knock down in 8% of dividing cells. Comparative immunofluorescence analysis of average integral intensity measurements of immunofluorescently labelled centrosomal ASPM was performed for twenty-five metaphase U2OS cells treated with either ASP1, ASP2 or GL3 siRNAs. In comparison to the average ASPM spindle pole signal measured in the GL3 treated metaphases, a significant decrease of 84.3% in the average integral intensity of ASPM signal at the spindle pole was calculated after 72 hr treatment with ASP1, and 55.2% after treatment with ASP2 (Table 1). Immunofluorescence and Western blotting could not detect a change in overall expression level of ASPM in interphase cells (Figure 3B,C), suggesting that ASPM is stabilised when associated with the nuclear matrix and is more open to degradation after nuclear envelope breakdown. We therefore chose to investigate the phenotype associated with knock down of ASPM at the spindle poles of dividing cells.

**Table 1:** Down regulation of ASPM in response to siRNA mediated ASPM depletion in metaphase U2OS cells.

	GL3	ASP1	ASP2
Average integral intensity (μm^2^)	14805	2318	6631
Knockdown of spindle pole expression (%)	0	84.3	55.2
p value		<0.001	0.005

Comparative immunofluorescence analysis, measured as average integral intensity, was performed on the immunofluorescent centrosomal ASPM signal of twenty-five metaphase U2OS cells treated with ASP1, ASP2 or GL3 siRNAs. In comparison to the average integral intensity of ASPM spindle pole signal measured in the GL3 treated metaphases, a significant decrease in the average integral intensity of ASPM signal at the spindle pole was calculated after 72 hr treatment with ASP1, and with ASP2. The knock down is expressed as a percentage of the expression in GL3 cells. In each case t-tests showed the levels of knockdown to be significant.

A substantial reduction in proliferation of U2OS cells was observed after siRNA treatment in a Celltitre Blue cell viability assay (Figure 3D). Assessment of the proportions of 2N:4N DNA content by flow cytometry 48 hours and 72 hours after *ASPM *siRNA treatment distinguished 4N cells in G2/M from tetraploid cells that had failed cytokinesis by confirming the presence or absence of Cyclin B1. We identified an increase in the proportion of 2N cells in G1, a reduction of cells in G2/M and an increase in tetraploid cells in *ASPM *siRNA treated cells (Figure 3E). These data are consistent with both a decrease in the number of cells undergoing mitosis and a failure of cytokinesis. This observation was corroborated by microscopic examination of immunofluorescently labelled cells after 48 and 72 hours of ASP1 and ASP2 siRNA knockdown, which indicated a decrease in mitotic index and an increase in multinucleate cells and large mononucleate cells with multiple centrosomes consistent with a failure to complete cytokinesis (Figure 3F). The number of cells in cytokinesis, defined in this instance as daughter cells linked by narrow cytoplasmic bridges containing midbodies, was also decreased by *ASPM *siRNA.

To gain further insight into the ASPM knockdown phenotype, we carried out phase contrast time-lapse imaging of living U2OS cells 72 hours post-ASP1 or GL3 siRNA treatment. The results of four independent experiments were combined to produce the final data set. We monitored 126 cell divisions of GL3 treated U2OS cells and 94 cell divisions following ASP1 siRNA. Depletion of ASPM had a profound effect on U2OS cell division. Three major phenotypic anomalies were identified; an increase in the proportion of divisions with the mitotic spindle perpendicular to the plate surface, an increase in cytokinesis failure, and an increase in apoptosis.

**Figure 4:**
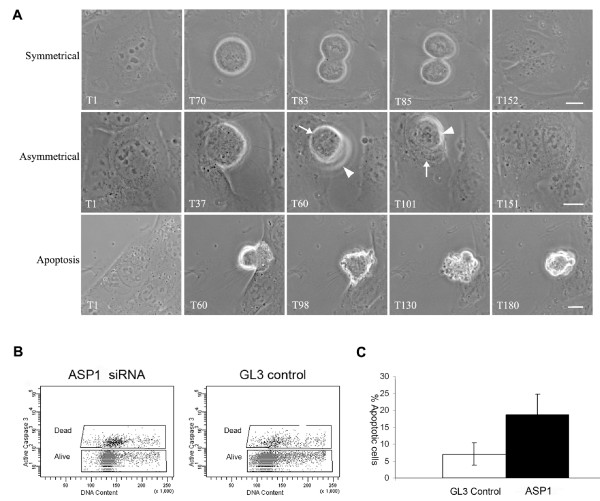
**siRNA mediated ASPM induces misorientation of the plane of cell division, cytokinesis failure and increased apoptosis in U2OS cells**. **A**. Panels showing phase contrast images of living U2OS cells taken from time-lapse movies. Time points are shown in the bottom left hand corner of each image. *Top row*. Normal symmetrical division of a GL3 siRNA treated control cell with the cleavage plane oriented perpendicular to the cell substrate (see Additional file 5). Scale bar = 10 μm. *Middle row*. ASP1 siRNA treated cell undergoing an asymmetric division with the mitotic spindle positioned perpendicular to the cell substrate. This division led to cytokinesis failure with the formation of a binucleate cell. Arrowhead shows the upper daughter cell moving out of the focal plane towards the observer as the cleavage furrow is formed parallel to the surface of the imaging dish. An arrow indicates the lower daughter cell still attached to the surface of the dish (see Additional file 6). Scale bar = 10 μm. *Bottom row*. Binucleated ASP1 siRNA treated cell undergoing apoptosis (see Additional file 7). Scale bar = 10 μm. **B**. Flow cytometric analysis of caspase-3 assayed 96 hours after ASP1 and GL3 siRNA treatment. An increase in the population of apoptotic (dead) cells is observed in ASP1 siRNA experiments. **C**. Histogram showing the mean percentage of apoptotic cells in ASP1 and GL3 siRNA caspase-3 assays. A 2.7 fold increase in the percentage of apoptotic cells in the ASP1 siRNA population was identified compared to the GL3 siRNA population. Standard deviation values for the mean are shown as vertical bars.

Normal symmetrical division necessitates positioning of the mitotic spindle parallel to the surface of the imaging dish resulting in cleavage plane orientation perpendicular to the dish (Figure 4A, see Additional file 5). In 37% of ASP1 siRNA knockdowns (n = 36) random deviation in the position of the mitotic spindle was observed (Table 2) with a significant increase in spindle positioning approximately perpendicular to the surface of the dish (Figure 4A, see Additional file 6). This spindle alignment is reminiscent of that observed in asymmetric divisions in mouse progenitor cells of the neuroepithelium [35]. We therefore termed these divisions asymmetric. In comparison, only 10.3% (n = 13) of GL3 treated cells exhibited such asymmetric divisions.

**Table 2:** Effect of siRNA mediated ASPM depletion on spindle pole position and cytokinesis.

	Total number of divisions	Symmetric division with cytokinesis (% of total)	Symmetric division with cytokinesis failure (% of total)	Asymmetric division with cytokinesis (% of total)	Asymmetric division with cytokinesis failure (% of total)
GL3	126	109 (86.5%)	4 (3.2%)	8 (6.3%)	5 (4.0%)
ASP1	94	52 (55.3%)	6 (6.4%)	9 (9.6%)	27 (28.7%)

Time-lapse imaging of living U2OS cells was performed 72 hours post-GL3 and ASP1 siRNA treatment. The results of four independent experiments were combined to produce the final data set. Depletion of ASPM had a profound effect on U2OS cell division, resulting in an increase in the proportion of divisions with the spindle positioned perpendicular to the plate surface (asymmetrical division) and an increase in cytokinesis failure.

A second consequence of ASPM depletion observed by time-lapse imaging was cytokinesis failure (Table 2), leading to the formation of multinucleated cells. Although we observed this phenomenon in a minor population of ASP1 siRNA-treated cells that underwent symmetrical division (6.4%, n = 6), the phenotype was far more prevalent in ASP1 treated cells that had undergone asymmetric divisions (28.7%, n = 27) (Figure 4A, see Additional file 6). In contrast, cytokinesis failure was observed in only 3.2% (n = 4) of symmetric cell divisions and 4% (n = 5) of asymmetric cell divisions in GL3 treated cells. In this culture system asymmetric cell division initially results in one daughter cell anchored to the culture dish with the second daughter cell positioned above the first (i.e., being extruded towards the observer see Additional file 6) and not attached to the substrate. We observed that in many instances the second daughter cell was able to subsequently make contact with the dish and successful cytokinesis occurred. Only 38% (5 of 13 cells) of asymmetrical divisions in GL3 control experiments failed cytokinesis compared with 75% (27 of 36 cells) of asymmetrical ASP1 treated cells. Therefore asymmetrical division per se does not fully account for increased cytokinesis failure in ASP1 siRNA treated U2OS cells.

Thirdly, a visible increase in cell death was observed in cells transfected with ASP1, 72 hours post transfection. Apoptosis was observed in mononucleate cells, binucleate cells (Figure 4A, see Additional file 7) and in cells exiting mitosis but exhibiting cytokinesis failure. To quantitate the extent of ASP1 siRNA induced apoptosis we performed an *in situ *activated caspase-3 assay on unfixed U2OS cells after 96 hours of siRNA treatment (Figure 4B). On average, ASP1 siRNA treated cells demonstrated a 2.7 fold increase in caspase-3 positive apoptotic cells compared to control GL3 siRNA cells (mean percentage of apoptotic GL3 siRNA cells in a total cell population = 7% (s.d. = 3.3%) compared to mean percentage of apoptotic ASP1 siRNA cells = 18.7% (s.d. = 6%) (Figure 4C).

It has previously been reported that ASPM is required for precise orientation of the mitotic spindle in specialised mouse NPC [35]. Our data demonstrates that ASPM function is a determinant of spindle position and division symmetry even in non-neuronal derived cells, suggesting that complete absence of ASPM function during human development might be expected to affect whole body development. Since this is not the observed phenotype in MCPH, the effect of *ASPM *mutation on the function of the ASPM protein expressed in MCPH patients was a logical progression of our studies.

### An ASPM *C*-terminal splice donor site mutation induces novel splicing and reduces mitotic spindle pole association

ASPM contains a number of potential functional domains. Following the positional cloning of the *ASPM *gene, the initial genotype-phenotype correlation studies in MCPH patients did not reveal a clustered distribution of microcephaly-associated mutations in *ASPM *[5,15] and there were therefore few clues to which of these domains were most important for ASPM function.

**Figure 5:**
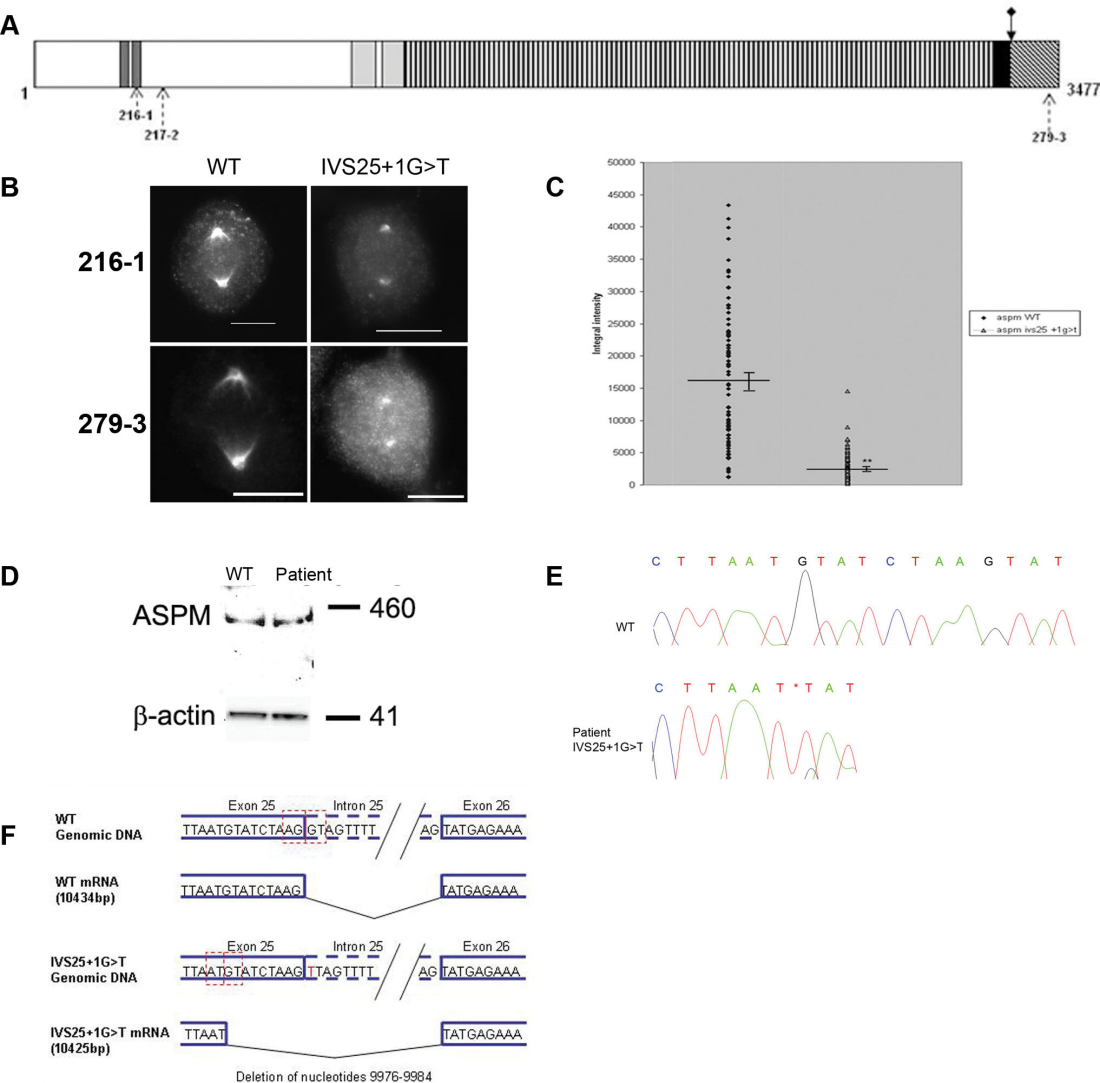
**ASPM^IVS25+1G > T ^mutation produces a novel splice variant with decreased efficiency for spindle pole localisation**. **A**. Schematic of the ASPM protein showing the location of the ASPM^IVS25+1G > T ^splice site mutation (filled arrows with an upper diamond). Domains of the protein are indicated as described in the legend to Figure 1. Location of epitopes of anti-ASPM antibodies are shown beneath the protein. **B**. Fibroblasts at metaphase immunostained using 216-1 anti-ASPM antibody (N-terminal) and the 279-3 anti-ASPM antibody, raised against a C-terminal peptide sequence. Note the reduced ASPM localization at the mitotic spindle poles of ASPM^IVS25+1G > T ^cells. Scale bar = 10 μm. **C**. Chart representing integral intensity of ASPM 216-1 antibody staining at the spindle poles of *ASPM*^*wt *^and *ASPM*^*IVS25+1G > T *^fibroblast cells. Horizontal lines indicate the average integral intensity of the immunostaining. Standard error bars are present. ASPM^IVS25+1G > T ^was at statistically significantly lower level (asterisks) at the spindle poles than ASPM^wt ^when compared using a paired two tailed t-test. P = < 0000.1. **D**. Immunoblotting of fibroblast control (WT) and *ASPM*^*IVS25+1G > T *^(Patient) lysates with 217-2 anti-ASPM antibody and anti β-actin. Equivalent levels of stable ASPM protein are expressed by each cell culture, indicating the IVS25+1G > T mutation does not induce nonsense mediated decay. **E**. Sequence analysis of exon 25-exon 26 control *ASPM*^*wt *^and Patient *ASPM*^*IVS25+1G > T *^cDNA. The point of removal of the nine nucleotides due to the IVS25+1G > T mutation is indicated by an asterisk. **F**. IVS25+1G > T mutation removes the exon 25 splice donor site and instigates the utilisation of an in frame splice donor site nine nucleotides downstream. The resultant ASPM^IVS25+1G > T ^protein is lacking just three amino acids (3326-3328).

We have previously characterized a *C*-terminal *ASPM *mutation as a homozygous intronic mutation at IVS25 +1G > T (9984 +1 G > T), identified in individuals of a consanguineous family of Northern Pakistani origin [15]. This mutation lies downstream of the Armadillo repeat-like domain (Figure 5A). Splice site prediction programs forecast the outcome of this mutation to be the removal of the intron 25 splice donor site, resulting in translational extension of exon 25 by 29 amino acids before the incorporation of a stop signal. We predicted this would result in either the synthesis of a truncated ASPM protein of 395 kDa (3357 amino acids) that would not include the anti-ASPM 279-3 peptide sequence, or that it could lead to ASPM depletion due to mRNA degradation via the NMD pathway.

To determine the effect of the IVS25+1G > T mutation on ASPM mitotic expression and localisation, comparative immunofluorescence microscopy of dividing *ASPM*^*wt *^and *ASPM *^*IVS25 +1G > T *^fibroblasts was performed using our *N*-terminal 216-1 anti-ASPM antibody. Quantitative analysis of spindle pole staining revealed a significant reduction in ASPM levels at the PCM in *ASPM *^*IVS25 +1G > T *^fibroblasts (Figure 5B, C) (n = 80, average integral intensity *ASPM*^*wt *^= 16427.01 μm^2 ^(s.e.m. = 1166.3 μm^2^), average integral intensity *ASPM*^*IVS25 +1G > T *^= 2325.1 μm^2 ^(s.e.m. = 260.1 μm^2^), p = < 0.0001; Average area *ASPM*^*wt*^= 4.80 μm^2^(s.e.m. = 0.27 μm^2^), average area *ASPM*^*IVS25 +1G > T *^^*t*^= 1.28 μm^2 ^(s.e.m = 0.08 μm^2^), p = < 0.0001). Unexpectedly, analysis of *ASPM *^*IVS25 +1G > T *^fibroblasts immunofluorescently labelled with the *C*-terminal anti-ASPM 279-3 antibody also revealed weak but clearly visible spindle pole staining (Figure 5B), suggesting the IVS25+1G > T mutation did not induce a protein truncation.

To investigate the effect of the IVS25+1 G > T *ASPM *mutation on the ASPM protein, we carried out immunoblotting of cell lysates from control (*ASPM*^*wt*^) and patient (*ASPM *^*IVS25 +1G > T*^) fibroblasts with the 217-2 *N*-terminal anti-ASPM antibody. Expression of full length ASPM (410 kDa) in *ASPM*^*wt *^cell lines was observed and a band of approximately similar size and equivalent intensity was detected in *ASPM *^*IVS25 +1G > T *^protein extracts (Figure 5D). Thus the IVS25 +1 G > T mutation does not instigate NMD or protein instability in fibroblasts. Due to the large size of both the full length ASPM and the predicted IVS25+1G > T truncated product (395 kDa) we were unable to directly determine size differences in the proteins by immunoblotting.

To further explore the effect of the IVS+1G > T mutation, RT-PCR of ASPM exons 24-28 of *ASPM*^*wt *^and *ASPM *^*IVS25 +1G > T *^fibroblast mRNA was performed and the single PCR product obtained from each was sequenced. Wildtype ASPM sequence was observed in the *ASPM*^*wt *^fibroblast mRNA. As expected the G > T mutation at the exon 25 splice donor site invalidated the native splice site sequence in the *ASPM *^*IVS25 +1G > T *^mRNA. However, transcription did not continue for the expected 29 amino acids before the incorporation of a STOP codon. Unexpectedly, an in-frame cryptic splice donor site nine base pairs upstream was recognised and utilised (Figure 5E, F). The use of the upstream cryptic splice donor site would result in the production of an ASPM protein from which only a tripeptide motif (amino acids 3326-3328) is deleted (Figure 5E). Our immunofluorescence data therefore indicates that the loss of three amino acids in the *C*-terminus of ASPM is apparently sufficient to drastically reduce the ability of the protein to localise with the microtubules at the PCM at the spindle poles. The fact that fibroblasts from this MCPH patient have a significant decrease in the quantity of microtubule associated ASPM suggests the presence of a functional *C*-terminal domain in ASPM that is critical for normal ASPM function.

### Expression of *C*-terminal fragments of ASPM inhibits spindle assembly and induces mitotic delay in HeLa cells

**Figure 6:**
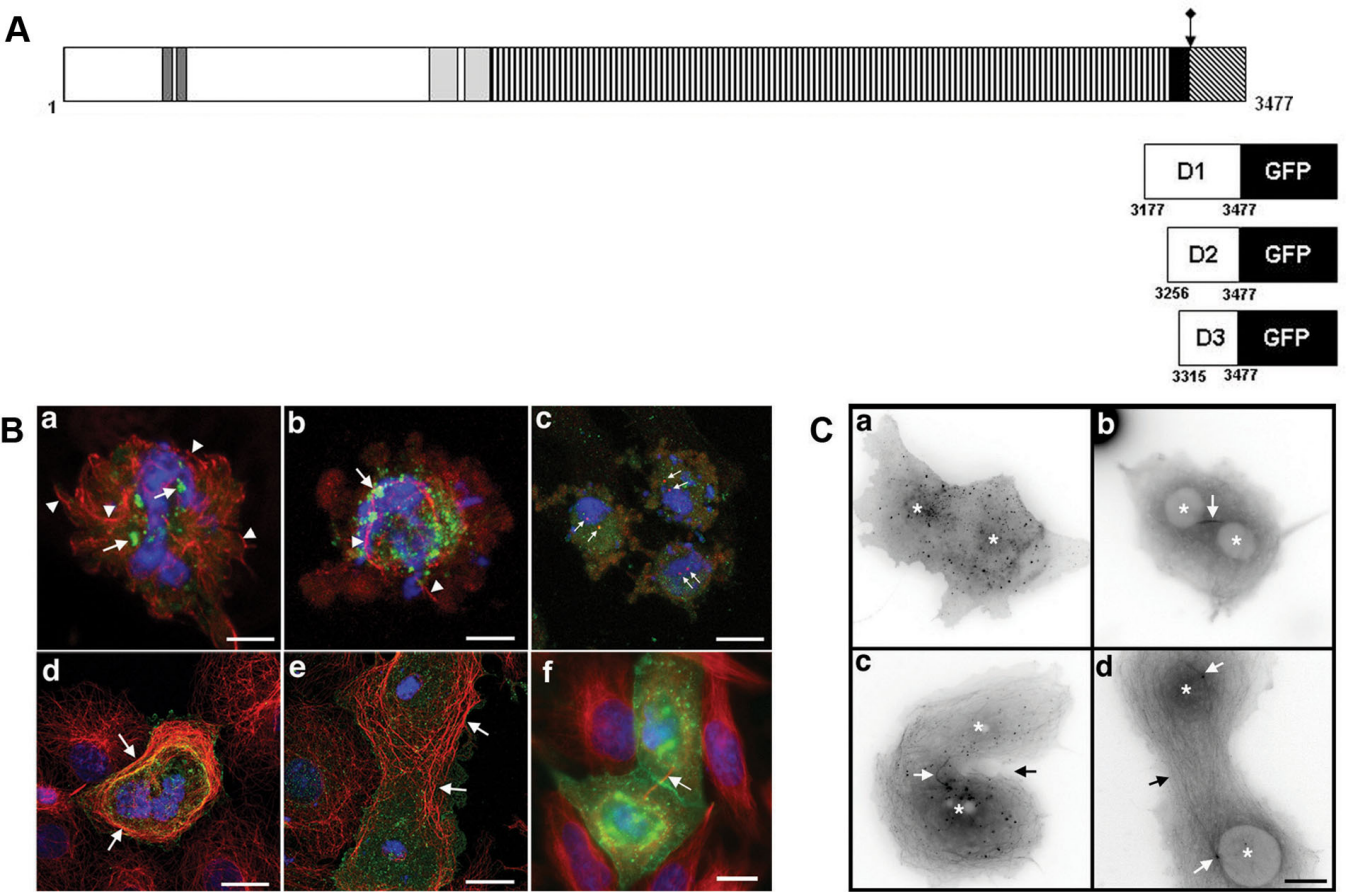
**Mitotic defects in transfected HeLa and COS-7 cells expressing *C*-terminal ASPM GFP fusion proteins**. **A**. Organisation of the 3477 amino acid human ASPM protein as described in the legend to Figure 1. *C*-terminal fragments expressed as fusion proteins with GFP are indicated D1-D3. Position of the *C*-terminal mutation (IVS25+1G > T) is indicated by an arrow. **B**. Panels a, b; HeLa cells expressing ASPM-D1-GFP immunostained to reveal GFP (green), α-tubulin (red) and DAPI (blue) then examined by confocal microscopy. Aggregates of fusion protein are indicated by arrows. Microtubules are disorganized and unfocused (arrowheads) and spindle poles cannot be identified. Scale bars = 5 μm. Panel c; HeLa cells expressing ASPM-D1-GFP immunostained to reveal GFP (green), γ-tubulin (red) and DAPI (blue). These abnormal cells possessed two spindle poles (arrows) and condensed DNA, suggesting mitotic arrest. Scale bar = 10 μm. Panels d-f; COS-7 cells expressing ASPM-D1-GFP immunostained to reveal GFP (green), α-tubulin (red) and DAPI (blue). Transfected cells were large and binucleate (panels e-f) or possessed abnormal lobular nuclei (panel d). Peripheral microtubule arrays or bundles were present (panels d-e, arrows). Panel f shows a binucleate cell containing a midbody (arrow). Scale bar = 20 μm. **C**. Aberrant cytokinesis in living COS-7 cells expressing ASPM-D2-GFP. Panel a; a binucleate COS-7 cell expressing ASPM-D2-GFP. Small aggregates of fusion protein can be seen. Panels b-d; COS-7 cells co-expressing ASPM-D2-GFP and EB3-GFP, used as a marker of microtubule organisation. Binucleate cells containing EB3-GFP positive midbodies (panels b and c, white arrows) or no midbody and a loose microtubule array (panel d, black arrow) are shown. Black arrow in panel c indicates the position of an incomplete and unusually asymmetrical cleavage furrow. White arrows in panel d indicate the position of centrosomes associated with the two nuclei, suggesting chromosomal segregation has occurred but cytokinesis has failed. In all panels asterisks indicate the location of nuclear structures. Images are shown in inverted grayscale for clarity. Scale bar = 10 μm.

Our immunostaining data in patient cells indicates a function mediated by the extreme *C*-terminal region of ASPM is required for its localization to spindle poles. We therefore examined the consequences for mitotic cells of expressing *C*-terminal fragments of ASPM upon mitotic progression. Plasmids directing the expression of GFP fused to three different fragments of 301 (D1), 222 (D2) and 163 (D3) amino-acids from the *C*-terminal sequence of ASPM were created (Figure 6A) to determine whether ectopic expression of these fragments would disrupt the function of endogenous ASPM in a dominant-negative manner.

HeLa cells were transfected with the plasmids and observed 24 hours post transfection. Each construct led to similar abnormalities that included the generation of a large number of cells with morphological features consistent with an early mitotic arrest (Figure 6B, panels a-c) and in many cells membrane blebbing was also evident (Figure 6B, panels b-c). The DNA within these cells was highly condensed and occasionally fragmented, while spindle microtubule organisation was severely disrupted. Where visible, microtubule focusing and spindle formation appeared to be fundamentally impaired (Figure 6B, panel a, arrowheads). In most cells the density of microtubules was decreased (Figure 6B, panel b, arrowheads). γ-tubulin immunostaining invariably revealed the presence of two separate foci in these cells (Figure 6B, panel c, arrows), consistent with the hypothesis that these were cells displaying a disrupted mitosis. Endogenous ASPM immunoreactivity was dispersed (not shown). This is consistent with the observation of diminished microtubule density and our earlier finding that the ASPM spindle pole association is microtubule-dependent. It is also consistent with the interpretation that expression of these ASPM fragments directly inhibited the localisation of endogenous ASPM to spindle poles. Of control cells expressing GFP alone, 3% (n = 245) displayed an apoptotic or mitotic delay phenotype, in comparison with 33% (n = 135) of cells expressing ASPM-D1-GFP; 26% (n = 127) of cells expressing ASPM-D2-GFP; and 17% (n = 131) of cells expressing ASPM-D3-GFP. Similar observations were obtained in transfected COS-7 cells (not shown). We conclude that a high proportion of HeLa cells expressing ASPM *C*-terminal fragments, particularly those containing the whole of the armadillo repeat-like region (D1 and D2), are defective in assembling a functional spindle. This leads to mitotic arrest and eventual apoptosis.

### Expression of ASPM *COOH*-terminal GFP-fusion proteins induces cytokinesis failure in COS-7 and HeLa cells

In addition to the severe mitotic defects seen in COS-7 and HeLa cells transfected with *C*-terminal fragments of ASPM, we also observed a subset of cells that appeared to have undergone mitosis but had failed to complete cytokinesis (Figure 6B, panels d-f). Defects ranged from cells containing large, highly lobular nuclei and peripheral microtubule bundles (Figure 6B, panel d, arrows) to cells containing discrete nuclei separated by aberrant microtubule arrays (Figure 6B, panel e, arrow), or structures resembling residual central spindles (Figure 6B, panel f, arrow). In neither case was there clear evidence for the presence of a cleavage furrow.

To exclude fixation artefacts we used co-expression of a GFP-tagged microtubule plus-end binding protein, EB3 [39,40] as a means of observing centrosomes and the tips of growing microtubules and midbody structures in living COS-7 cells. Expression of low levels of EB3-GFP alone had no significant impact on cell division in transfected COS-7 cells (J. M. Askham, unpublished data, 41). The phenotypes of COS-7 cells co-expressing ASPM-D2-GFP and EB3-GFP confirmed our immunostaining observations (Figure 6C). Expression of ASPM-D2-GFP alone led to the formation of binucleated cells (Figure 6C panel a). Co-expression of EB3-GFP revealed that these cells contained central spindle structures or loose parallel microtubule arrays (Figure 6C, panel b-d). In some examples centrosomes were seen in close association with well-separated nuclei (Figure 6C, panel d), indicating that the primary defect in these cells was a failure in cytokinesis after the completion of chromosomal segregation. The observations from both fixed and living COS-7 cells were therefore consistent with a role for ASPM in organizing the central spindle microtubule array in mitotic cells from anaphase onwards.

## Conclusions

Our study provides a mechanism by which mutations in the human *ASPM *gene could result in a developmental reduction of brain size, and supports the hypothesis that the MCPH phenotype arises due to defective NPC division. By examining the consequences of siRNA mediated knockdown of spindle pole associated ASPM expression and the phenotypes seen following expression of fusion proteins derived from the ASPM *C*-terminus we have identified critical roles for human ASPM in spindle microtubule organisation, spindle positioning and in the regulation of cytokinesis even in a non-neural cell type. Similar phenotypes have been previously reported in *Drosophila asp *mutants [25,28-33]. We suggest that ASPM is a reasonably stable protein and that nuclear matrix associated ASPM is less open to degradation than ASPM after nuclear envelope breakdown. ASPM was recently confirmed as a gene involved in the regulation of mitosis in human cells as part of the substantive MitoCheck integrated research project ([42]http://www.mitocheck.org). As an element of this high-throughput whole genome RNAi screen, automated live cell image analysis of ASPM knockdown identified similar phenotypes to those identified in our study. Although mitotic spindle position was not a phenotype for which analytical parameters were specifically established, other mitotic phenotypes that could lead to a decrease in cell division were identified. Metaphase delay was robustly observed with further phenotypes of mitotic delay, problems with metaphase alignment, cell death and poly-lobed nuclei identified with one of two siRNAs utilised.

We have previously shown that *Aspm *is preferentially expressed during cerebral cortical neurogenesis in the mouse brain [5]. The time of maximal *Aspm *expression in the neuroepithelium corresponds to the period of proliferative cell division, and the subsequent down-regulation of *Aspm *expression is concomitant with a switch to asymmetrical cell division [35]. We have now demonstrated that inhibition of ASPM function by siRNA causes a highly penetrant loss of precision in the placement of the mitotic spindle in dividing U2OS cells, resulting in an alteration of division mode from the symmetrical to the asymmetrical plane. This implies that in U2OS cells symmetrical cell division in a plane that is perpendicular to the substrate is an active process that requires functional ASPM, rather than a simple default pathway. Our data therefore demonstrates that the requirement for ASPM in the maintenance of symmetrical divisions is not limited to specialised NPCs as previously assumed, but that it plays a general role in mitotic cells.

A logical consequence of this hypothesis is that the normal development of the whole human body should be affected by ASPM loss of function. Indeed in *Drosophila *loss of asp function results in larval lethality [25,28-33]. Paradoxically however, the only known phenotype in individuals with homozygous mutations in *ASPM *is MCPH [5,15]. Mutations in *ASPM *are scattered throughout the gene, yet they result in a single clinical MCPH phenotype. Our immunoblot data of ASPM expression in fibroblast lysates from an MCPH patient carrying the IVS25+1G > T homozygous mutation established that the MCPH phenotype in this patient did not result from complete ASPM loss and confirmed a previous report that *ASPM *mutation does not instigate the NMD pathway [8]. Partly functional ASPM proteins may therefore be expressed in the cells of MCPH individuals, and we hypothesised that *ASPM *mutations caused protein truncations and that the MCPH phenotype resulted from the common loss of a *C-*terminal functional domain that led to partial loss of ASPM function. Surprisingly, our data from patient cells demonstrates that the MCPH phenotype can arise from a cryptic splicing event that removes only nine nucleotides of intragenic sequence within the *C*-terminal region of ASPM and results in a reduction in ASPM localisation to the spindle poles. The minimal IVS25+1G > T mutation therefore causes a decrease in the efficiency of ASPM spindle pole localisation without an associated decrease in overall ASPM protein levels or a major increase in gross mitotic abnormalities in patient fibroblasts. We propose that expression of this subtly impaired mutant ASPM protein is sufficient to induce small deviations in the precision of mitotic spindle positioning in symmetrically dividing cells. This contrasts with the severe mitotic phenotypes seen in cultured cells following more profound disruption of ASPM function by siRNA knockdown of ASPM spindle pole expression, or expression of dominant negative ASPM *C*-terminal domains.

The existence of individuals homozygous for mutations in *ASPM *who exhibit a small brain but who are otherwise grossly normal leads us to infer that either (a) a functional compensatory mechanism exists in somatic cells or (b) neurogenic cell divisions are sufficiently different to the majority of somatic cell divisions to be profoundly and differentially affected by a subtle perturbation of ASPM function. The reason for this may lie in the unique morphology of the cells undergoing division in the neuroepithelium during cortical expansion [35]. In vertebrates, apical NPC are apicobasally elongated to a remarkable extent and possess a small apical surface. To execute accurate symmetrical divisions in such cells would require extremely precise cleavage along the apicobasal axis [43]. Small deviations in spindle position leading to cleavage plane reorientation would result in a transition from symmetrical to asymmetrical division. In the majority of somatic cells such deviations could be tolerated due to their relatively large apical and basal surfaces. However in NPC such deviation may be sufficient to drive a decrease in the number of cells successfully completing symmetrical cell division. This would impair the expansion of the progenitor pool that normally occurs at early stages of cortical development. As a consequence, the NPC pool would be insufficient to produce the number of neurones required for a normal sized brain.

What then is the function of ASPM during the later stages of mitosis? We observed an ASPM localisation at the minus ends of central spindle microtubules during anaphase and at the centre of the midbody during telophase and cytokinesis. Microtubules of the mitotic apparatus are a critical contributor to cleavage furrow positioning. Both astral microtubules and overlapping equatorial MTs in the central spindle have been implicated as playing a significant role in this process [44-47]. We therefore see a number of possibilities for ASPM function during cytokinesis. ASPM might directly contribute to the organisation of midzone microtubules with the cytokinesis defects seen after ASPM functional inhibition arising secondary to central spindle disorganisation. Alternatively, its presence at the centrosome might influence the function of astral microtubules. It also seems possible that ASPM might participate more directly in the coupling of spindle microtubule function to cortical events during furrowing. For example, ASPM might be able to influence the local activity of myosins through its interactions with EF-hand Ca2+ binding factors such as calmodulin, or to influence signalling events during cytokinesis through interactions with binding partners such as citron kinase [38].

In conclusion, a major finding of our study is that ASPM plays a role in cell division, not just those in NPCs, but in other cell types. We propose that *MCPH5 *patients have enough residual ASPM activity to successfully complete functionally symmetrical cell divisions in all tissues except the developing brain, where extremely unusual morphological constraints result in a specific defect in cortical neurogenic mitosis in response to imprecise spindle position. In this we echo the arguments presented by Fish *et al*., in an authoritative commentary on NPC division [43]. We have also identified a three amino acid sequence in the *C*-terminal domain of ASPM in an MCPH patient reduces localisation of ASPM to the PCM implying that this region of ASPM mediates an important function in NPCs. Future studies will be aimed at defining whether this region of ASPM mediates an important interaction with a novel binding partner.

## Methods

### Cell culture

HeLa and COS-7 cells were obtained from the European Collection of Cell Cultures. U2OS human osteosarcoma cells and SH-SY5Y neuroblastoma cells were obtained from the ATCC Cell Biology Collection (ATCC-LGC Promochem Partnership, South London, UK). Human neonatal dermal fibroblasts (*ASPM *^*wt*^) were obtained from Genlantis (San Diego, California). Each cell line was maintained in accordance to manufacturer's guidelines.

The *MCPH5 *patient fibroblast cell culture (A*SPM*^*IVS25+1G > T*^) was created from a forearm skin biopsy. The collection of biopsy sample was approved by the Ethical Committee of Leeds (East) Research Ethics Committee (REC reference number 05/Q1206/80) and informed consent was collected from the subject and parents. The skin biopsy was finely cut and homogenised to desegregate the cells prior to plating in Hams F10 medium containing L-glutamine and 25 mM HEPES (Invitrogen, Paisley, U.K.), 20% fetal calf serum (Invitrogen) and 1% penicillin/streptomycin (Invitrogen) and incubated in 5% CO_2 _at 37°C

### Antibodies and Cytotoxic reagents

Rabbit polyclonal antibodies against peptide sequences corresponding to aa363-386 (sequence code 216-1; KDNYGLNQDLESES), aa426-441 (sequence code 217-2; PEDWRKSEVSPRIPEC) and aa3443-3458 (sequence code 279-3; SRLKPDWVLRRDNMEE) of the human ASPM sequence were raised by Affiniti Research Products Limited, Exeter, UK. BLAST analysis revealed no significant homology to other published sequences. Antibody specificity in immunostaining was demonstrated by peptide pre-competition of immune serum and lack of specific staining from pre-immune serum (not shown). A rat anti α-tubulin antibody was obtained from Serotec Ltd, Oxford, UK. A mouse monoclonal antibody specific for γ-tubulin was obtained from the Sigma-Aldrich Company, Dorset, UK. Mouse monoclonal and rabbit polyclonal anti-GFP antibodies were obtained from Clontech (Saint-Germain-en-Laye, France). Secondary antibodies were Molecular Probes highly cross-absorbed rabbit, mouse or rat IgG-specific Alexa conjugates (Cambridge BioScience, Cambridge, UK). DAPI, nocodazole and taxol were obtained from the Sigma-Aldrich Company.

### Immunofluorescence

Cells were cultured on glass coverslips. Transfections of GFP-constructs were performed using GeneJuice (Novagen) according to the manufacturer's instructions and coverslips were processed for immunocytochemistry 24 hours after transfection. Cells were fixed for 5 minutes in ice-cold methanol and labelled as previously described [48]. Immunofluorescence analysis was performed using a Leica TCS-SP confocal microscope as described previously [49] and a Zeiss Axiovert 200 inverted microscope coupled to an Orca II ER CCD camera controlled by AQM6 software (Kinetic Imaging, Wirral, UK) as described previously [50]. All confocal images presented here are maximum intensity projections. Figures were assembled and annotated using Adobe Photoshop CS2.

### Immunoblotting

To perform immunoblotting of ASPM, cells were lysed in standard RIPA buffer, protein concentration determined and PAGE carried out using 3-8% Tris Acetate precast gradient gels (Invitrogen) in a Surelock electrophoresis tank (Invitrogen) on 25 μg protein. Subsequent protein transfer onto PVDF membrane (Invitrogen) was performed in the same system in accordance with the manufacturer's instructions.

### siRNA knockdown of ASPM expression

Double-stranded RNA oligos that target *ASPM *mRNA were obtained from Dharmacon Inc Lafayette, CO, USA. siRNA ASPM1 targets sequence UGCCAUGGUGCAACUUGCU (nt 691-709) (synthetic oligos UGCCAUGGUGCAACUUGCUUU and AGCAAGUUGCACCAUGGCAUU) and siRNA ASPM2 targets GUGGUGAAGGUGACCUUUC (nt 2804-2822) (synthetic oligos GUGGUGAAGGUGACCUUUCUU and GAAAGGUCACCUUCACCACUU). A GL3 siRNA targeting the firefly luciferase sequence CUUACGCUGAGUACUUCGA was used as a control [51].

5 × 10^5 ^U2OS cells per well of a six well plate containing a coverslip or 4 × 10^5 ^U2OS cells per 35 mm plate for live cell imaging experiments were transfected 16 hours post plating with siRNA using Oligofectamine transfection reagent (Invitrogen). siRNA transfection mix was prepared for each plate as follows; 12 μl of 20 μM siRNA was added to 200 μl Optimem serum free medium (Invitrogen). 8 μl of Oligofectamine was added to 52 μl Optimem medium in a separate tube and incubated at room temperature for 10 minutes. The diluted transfection reagent was added to the tube containing the diluted siRNA and incubated for a further 20 minutes. 128 μl of Optimem was then added to give a final siRNA transfection mix volume of 0.4 ml. The growth medium was removed from the cells which were washed once with growth medium without serum or antibiotics. 0.6 ml growth medium without serum or antibiotics was added to the plate, followed by the 0.4 ml transfection mix. The transfection was incubated at 37°C for 4 hours before adding 1 ml growth medium containing 20% FCS and antibiotics. The cells were incubated for a total of 48 or 72 hours before analysis.

### FACS analysis of DNA content

48 and 72 hours after siRNA treatment U2OS cells were released by trypsin digestion, pelleted, washed in PBS and resuspended in 1 ml of cold 70% ethanol then stored at -20°C. Prior to Flow Cytometric analysis the cells were washed in block buffer (PBS containing 1% BSA), permeabilised for 15 minutes in block buffer containing 0.25% Triton X-100 and then incubated for 1 hour in 100 μl of mouse anti cyclin B1 (BD-Pharmingen 554177 diluted 1:500). Cells were washed in block buffer then incubated for 1 hour with Alexa Fluor 488 anti-mouse IgG (Molecular probes A-11029, diluted 1:300). Cells were finally washed and then incubated at 37°C for 30 min in 0.5 ml PBS containing 3 μl of 6 mg/ml RNAse and 12.5 μl of 5 mg/ml propidium iodide. 10,000 single cell events per sample were analysed for DNA and cyclin B1 content to identify G1 (2N DNA content, low cyclin B1), G2/M (4N DNA content, high cyclin B1) and tetraploid cells (4N DNA content, low cyclin B1) using a Facscalibur Facscan (Becton Dickinson).

### CaspGLOW™ Fluorescein Active Caspase-3 Assay

U2OS cells were treated with GL3 or ASP1 siRNA duplex for 96 hours prior to commencing Caspase-3 analysis. Detached and adherent cells were collected and washed with PBS. The CaspGLOW™ Fluorescein Active Caspase-3 assay (Medical and Biological Laboratories Ltd, Woburn, MA, USA) was performed on 3 × 10^5 ^GL3 and ASP1 treated cells as per manufacturer's protocol. The cells were then resuspended in PBS containing 50 ug/ml propidium iodide, 100 ug/ml RNAse A and 0.1% (v/v) Triton X-100 and incubated at 37°C for one hour. Active caspase-3 was detected by flow cytometry using a BD LSRII (Becton Dickinson) capturing 10000 events per sample. Three independent experiments were performed

### Time-lapse live cell imaging

Phase contrast time-lapse imaging experiments of live cells were performed on a Zeiss Axiovert 200 M inverted microscope incorporating an automated motorised stage, contained within a Perspex incubation chamber heated to a constant 37°C temperature (Solent Scientific, UK). Cells were cultured, transfected and imaged in 35 mm glass-bottomed culture dishes (Iwaki brand; Asahi Techno Glass Corporation, Japan, obtained from Barloworld Scientific, UK). Growth media was replaced with 2 ml media supplemented with 20 mM HEPES. Cells were observed under oil with an ×40 Plan Neofluar 1.3 aperture objective. Images were captured with a Hamamatsu Orca Camera, using Volocity 3 acquisition software (Improvision, Coventry, UK) over a period of 4 hours. Multiple positions were imaged at a rate of one image per minute. Live cell fluorescence imaging experiments were performed on a Zeiss Axiovert 200 inverted microscope contained within a Perspex incubation chamber heated to a constant 37°C temperature (Solent Scientific, UK). Imaging was performed as described previously [50]. Figures were assembled and annotated using Adobe Photoshop CS2.

### Celltitre Blue cell viability assay and assessment of mitotic abnormalities in fixed immunolabelled siRNA treated cells

To perform cell viability assays on siRNA treated cells, U2OS cells were plated in 96-well plates at 10^3 ^cells per well and grown at 37°C for 16 hours before siRNA treatment (as above). Wells were washed once with serum free medium and then 60 μl serum free medium and 40 μl siRNA transfection mix were added. The transfection was completed as described above. 72 hours after siRNA transfection, Celltitre Blue reagent (Promega) was added to wells following manufacturer's instructions. The plate was incubated at 37°C for a further 8 hours and the conversion of resazurin to fluorescent resorufin, an assay of metabolic rate, was determined using fluorimetry (560 nm excitation wavelength, 615 nm emission wavelength). In parallel experiments siRNA treated and control cell cultures were co-immunostained using combinations of antibodies that included α-tubulin, γ-tubulin and ASPM. DNA was counterstained using DAPI. At least 1000 total cells per sample were then assessed by direct microscopic observation to determine mitotic index, the number of cells in cytokinesis, and the proportion of cells with abnormal numbers of nuclei and centrosomes.

### Comparative analysis of ASPM spindle pole expression in *ASPM*^*wt*^, *ASPM *^*IVS25 +1G > T *^cell lines and GL3, ASP1 and ASP2 treated U2OS cells

To compare relative quantities of ASPM at the spindle poles of *ASPM*^*wt *^and *ASPM *^*IVS25 +1G > T *^cells, cells were simultaneously immunofluorescently labelled using 216-1 anti-ASPM antibody. Images were captured on an Olympus BX61 upright microscope using ×100 oil UPlanFLN 1.30 aperture objective, F-View II monochromatic camera (Olympus Soft Imaging Solutions Ltd, Helperby, North Yorkshire, U.K.) and Cell^P ^Software (Olympus Soft Imaging Solutions Ltd), with a 1 second exposure time, ensuring the signal intensities visualised in the images were directly comparable. ASPM PCM signal intensity was measured as integral intensity (defined as the sum of all the intensities of a region of interest multiplied by the pixel area). To compare the integral integrity values of ASPM localised at the spindle pole in *ASPM*^*wt *^and *ASPM*^*IVS25 +1G > T *^cells a paired two tailed Student *t*-test was used. A *P*-value of less than 0.0001 was considered highly statistically significant.

To compare the integral integrity values of ASPM localised at the spindle pole in GL3, ASP1 and ASP2 treated metaphase U2OS cells captured with a 2 second exposure time, a Mann Whitney test was used. A *P*-value of less than 0.005 was considered statistically significant.

### RNA preparation, reverse transcription and sequencing

RNA was obtained from *ASPM*^*wt *^and *ASPM*^*IVS25 +1G > T *^fibroblasts using Trizol extraction as per manufacturers instructions. cDNA was produced using Superscript reverse transcriptase (Stratagene Agilent Technologies, West Lothian, UK) as per manufacturers direction with oligo dT primers (Promega). *ASPM *exons 25-28 were amplified using forward (TCCGAAGTTGTAATCGCAGT) and reverse (CTTGCAGGGGATTTGTGATT) primers.

### Plasmid construction

The plasmids pASPM-D1-GFP, pASPM-D2-GFP and pASPM-D3-GFP were constructed by cloning nt 9530-10434 (aa3177-3477), 9766-10434 (aa3256-3477) and 9943-10434 (aa3315-3477) of *ASPM *respectively in frame into the *Bam*HI and *Xho*I restriction sites in the GFP expression plasmid pJMA2-eGFP [49]. All constructs were fully sequenced before use. The construction and characterization of an EB3-GFP expression plasmid will be described elsewhere (J. M. Askham, manuscript in preparation).

## Abbreviations

ASPM: Abnormal spindle-like microcephaly associated; CDK5RAP2: CDK5 regulatory subunit-associated protein 2; CENPJ: Centromeric protein J; DCNT1: Dynactin1; FACS: Fluorescence-activated cell sorter; GFP: Green fluorescent protein MCPH: Autosomal recessive primary microcephaly; NPC: Neural progenitor cell; NMD: Nonsense mediated decay; nt: Nucleotide; PBS: Phosphate buffered saline; s.d.: Standard deviation; s.e.m.: Standard error of the mean; siRNA: Small interfering RNA

## Authors' contributions

Project conceived and experiments designed by JB, EEM, CGW, CAM, SMB and DMG. Experiments carried out by JB, JH, CAM, A-MB, RKB, SMB, and JMA. Patients diagnosed, recruited and tissue biopsies obtained by CGW, SMS and CB. Manuscript prepared by JB, EEM, CGW, CAM and DMG. All authors have read and approved the final manuscript.

## Supplementary Material

Additional file 1 **A comparison of ASPM interphase localisation in HDF cells using ASPM *N*- and *C*-terminal ASPM antibodies**. Cells were fixed and stained with the *N*-terminal ASPM antibody 216-1 or 217-2 or the *C*-terminal antibody 279-3 (green), anti-α-tubulin (red) and DAPI (blue) to identify nuclei. Scale bar = 10 μm. Click here for file

Additional file 2 **A comparison of ASPM metaphase localisation in HDF cells using ASPM *N*- and *C*-terminal ASPM antibodies**. Cells were fixed and stained with the *N*-terminal ASPM antibody 216-1 or 217-2 or the *C*-terminal antibody 279-3 (green), anti-α-tubulin (red) and DAPI (blue) to identify nuclei. Scale bar = 10 μm. Click here for file

Additional file 3 **ASPM is localised at the spindle poles in metaphase cells for a range of cell types**. HeLa, COS-7, U2OS, SH-SY5Y and HDF cells were fixed and stained with the *N*-terminal ASPM antibody 216-1 (green), anti-α-tubulin (red) and DAPI (blue) to identify nuclei. Scale bar = 10 μm. Click here for file

Additional file 4 **ASPM is positioned in a narrow ring at the centre of the midbody during telophase in a range of cell types**. Cells were fixed and stained with the *N*-terminal ASPM antibody 279-3 (green), anti-α-tubulin (red) and DAPI (blue) to identify nuclei. Scale bar = 10 μm. Click here for file

Additional file 5 **Symmetrical division in GL3 luciferase control siRNA treated cells**. A U2OS cell treated with GL3 siRNA undergoing symmetrical division with the mitotic spindle parallel to the surface of the imaging dish. This division results in two daughter cells and complete cytokinesis. Click here for file

Additional file 6 **Asymmetrical division and cytokinesis failure in a siRNA mediated ASPM depleted U2OS cell**. A U2OS cell treated with ASP1 siRNA undergoing an asymmetric division with the mitotic spindle perpendicular to the surface of the imaging dish. This results in one daughter being extruded vertically from the dividing cell, towards the observer, while the other remains attached to the substrate. In this example cytokinesis fails, resulting in the formation of a binucleate cell. Click here for file

Additional file 7 **ASPM depleted binucleate U2OS cell undergoing apoptosis**. This movie shows a binucleate U2OS cell following treatment with ASP1 siRNA, undergoing apoptosis. Click here for file
