# Supplementary figures and images for: Human ASPM participates in spindle organisation, spindle orientation and cytokinesis
Source: BMC Cell Biol. 2010 Nov 2;11:85. doi: 10.1186/1471-2121-11-85 (PMC2988714; doi:10.1186/1471-2121-11-85)

DNA

 $\alpha$ -tubulin

ASPM

Merged

216-1

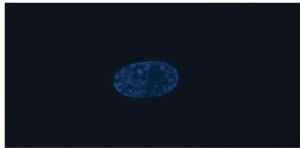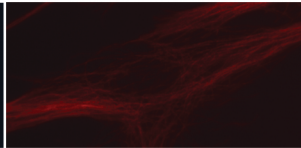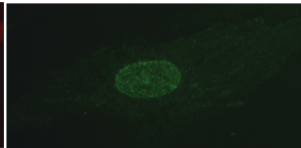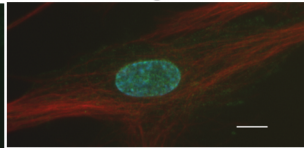

217-2

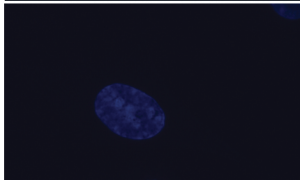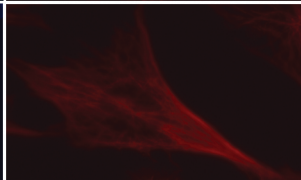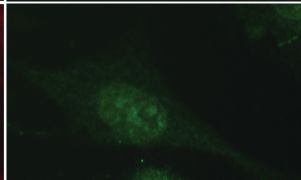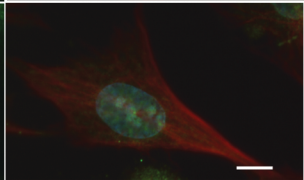

279-3

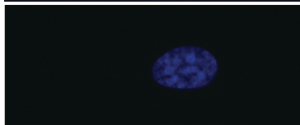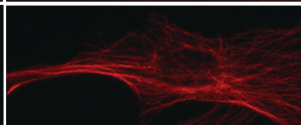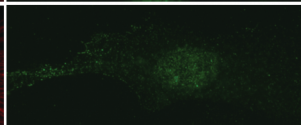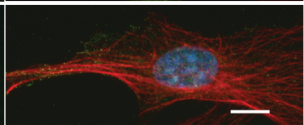

Supplement: Additional file 1 — A comparison of ASPM interphase localisation in HDF cells using ASPM N- and C-terminal ASPM antibodies. Cells were fixed and stained with the N-terminal ASPM antibody 216-1 or 217-2 or the C-terminal antibody 279-3 (green), anti-α-tubulin (red) and DAPI (blue) to identify nuclei. Scale bar = 10 μm. [file 1471-2121-11-85-S1.PDF]

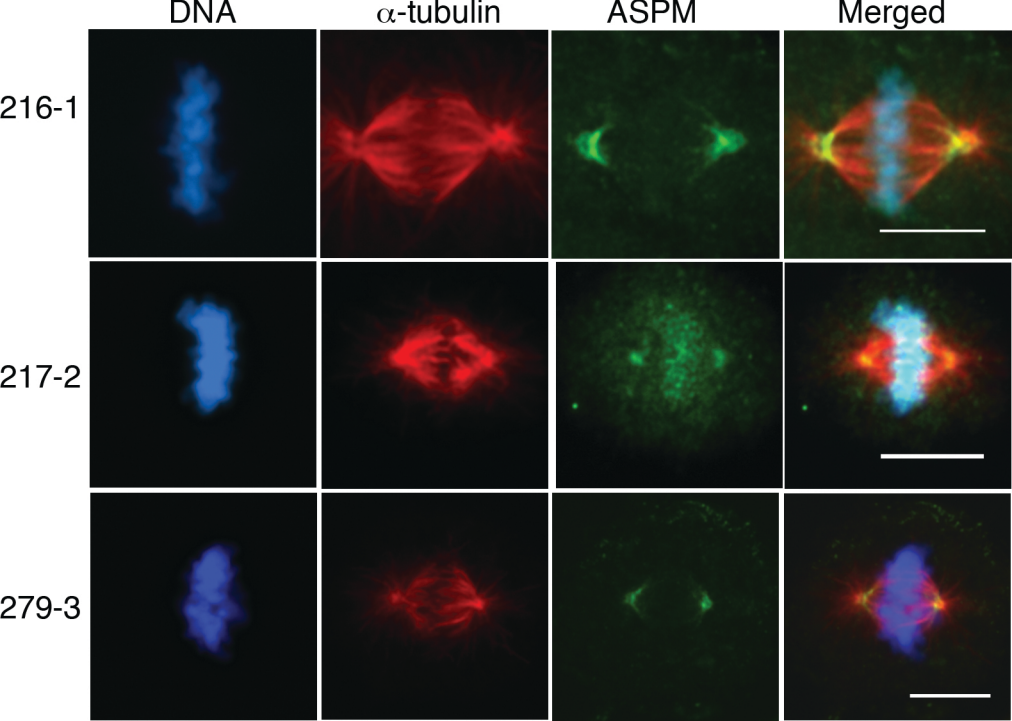

Supplement: Additional file 2 — A comparison of ASPM metaphase localisation in HDF cells using ASPM N- and C-terminal ASPM antibodies. Cells were fixed and stained with the N-terminal ASPM antibody 216-1 or 217-2 or the C-terminal antibody 279-3 (green), anti-α-tubulin (red) and DAPI (blue) to identify nuclei. Scale bar = 10 μm. [file 1471-2121-11-85-S2.PDF]

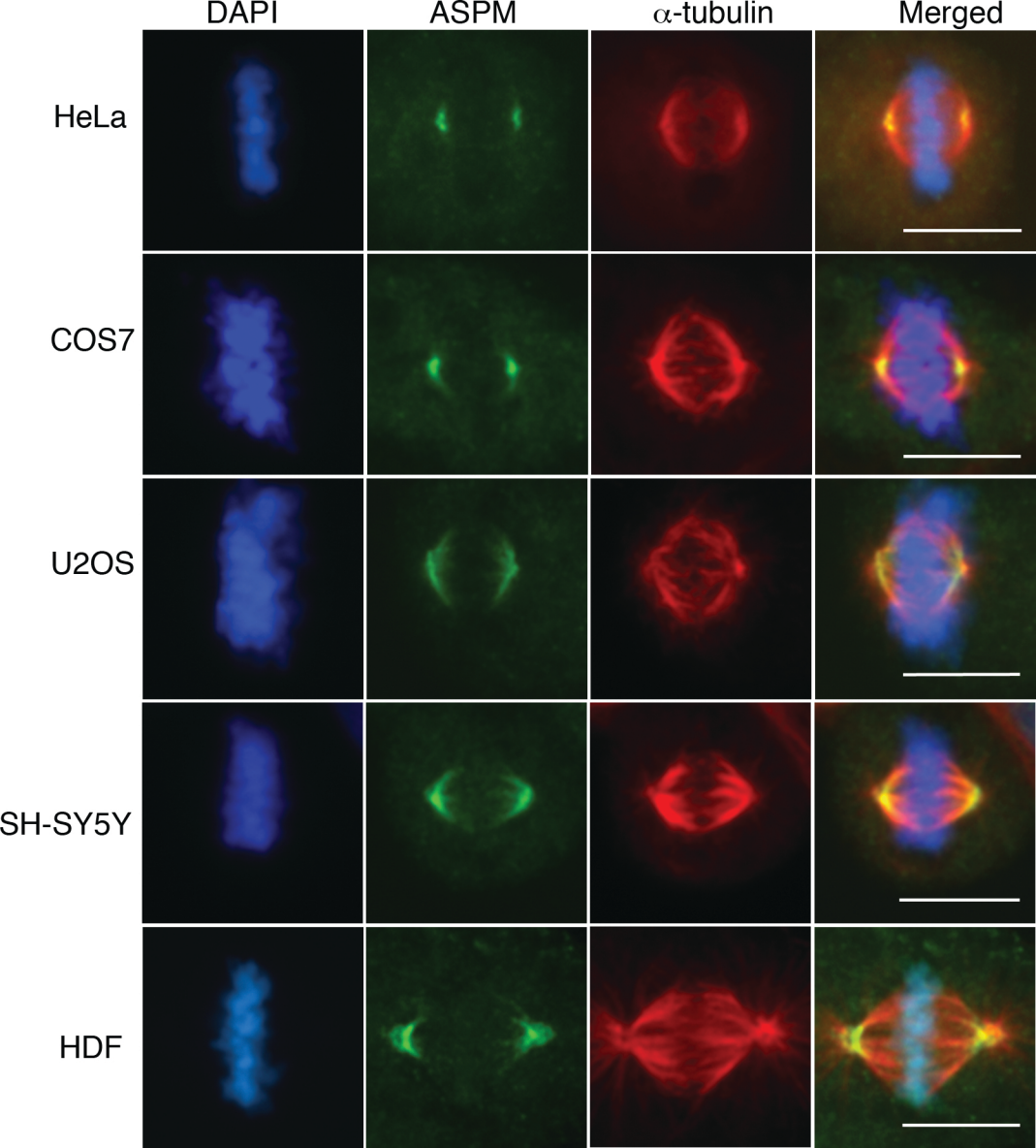

Supplement: Additional file 3 — ASPM is localised at the spindle poles in metaphase cells for a range of cell types. HeLa, COS-7, U2OS, SH-SY5Y and HDF cells were fixed and stained with the N-terminal ASPM antibody 216-1 (green), anti-α-tubulin (red) and DAPI (blue) to identify nuclei. Scale bar = 10 μm. [file 1471-2121-11-85-S3.PDF]

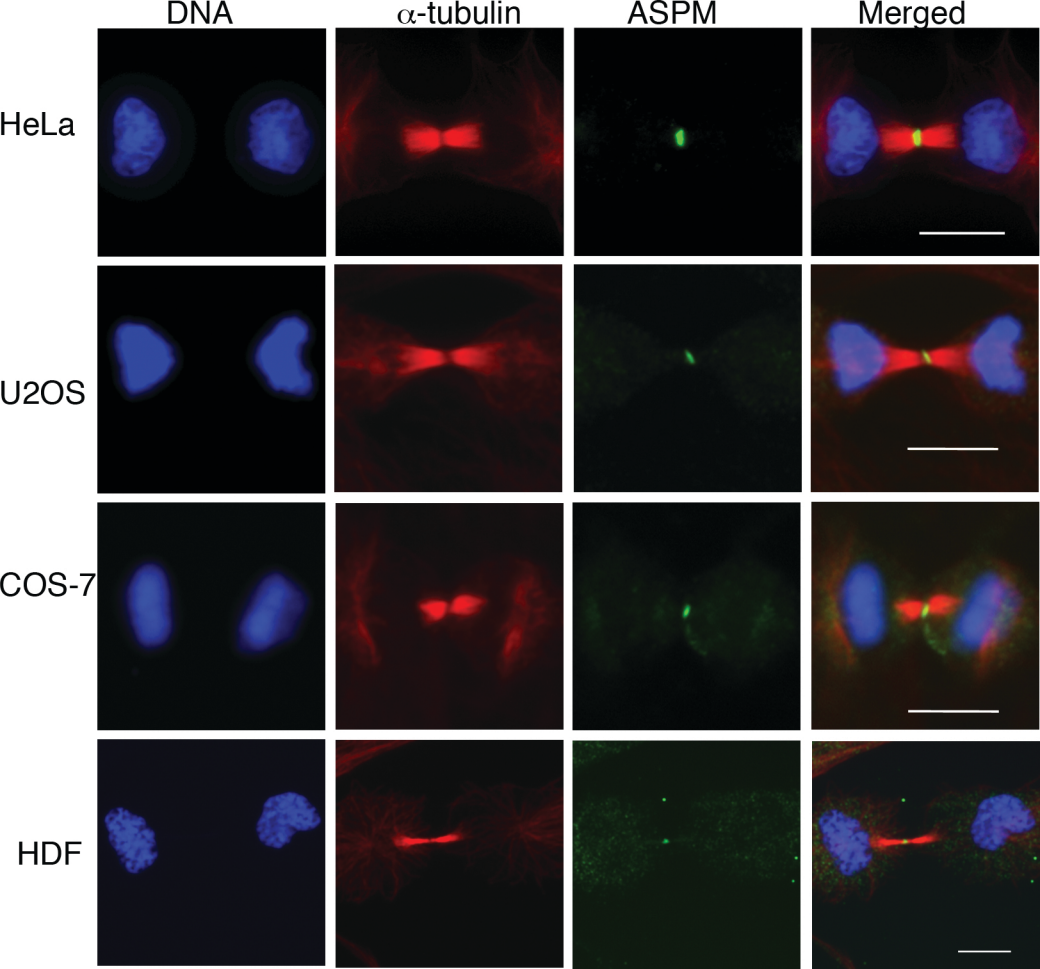

Supplement: Additional file 4 — ASPM is positioned in a narrow ring at the centre of the midbody during telophase in a range of cell types. Cells were fixed and stained with the N-terminal ASPM antibody 279-3 (green), anti-α-tubulin (red) and DAPI (blue) to identify nuclei. Scale bar = 10 μm. [file 1471-2121-11-85-S4.PDF]
